# An investigation of genetic diversity in three Dezhou donkey original breeding farms

**DOI:** 10.1038/s41598-023-38219-1

**Published:** 2023-07-11

**Authors:** Tianqi Wang, Ziwen Liu, Xiaoyuan Shi, Zhenwei Zhang, Yuhua Li, Bingjian Huang, Wei Ren, Xinrui Wang, Changfa Wang, Wenqiong Chai

**Affiliations:** Liaocheng Research Institute of Donkey High-Efficiency Breeding and Ecological Feeding, Agricultural Science and Engineering School, Liaocheng University, Liaocheng, 252059 China

**Keywords:** Genetics, Molecular biology

## Abstract

Dezhou donkey is one of the excellent large donkey breeds in China. In our study, eight microsatellite markers were used to genotype from each of three populations of Chinese Dezhou donkeys: 67 individuals in Liaocheng (pop1), 103 individuals in Binzhou 1 (pop2), and 102 individuals in Binzhou 2 (pop3), in order to study the genetic diversity of these populations. A total of 213 alleles were detected, and the *PIC* results showed that eight loci were all highly polymorphic. The means of *Ho* and *He* of pop2 were the highest, which were 0.646 and 0.717, respectively. The PCoA analysis demonstrated that the samples from three conservation farms were mixed together. The phylogenetic tree showed that pop 2 and pop 3 were closely related. The phylogenetic tree results clustered that 272 donkeys were divided into six groups. AMOVA analysis showed that the genetic variation was mainly concentrated within the population and the genetic differentiation among the populations was low. Fst values between populations also indicated that genetic differentiation between populations was too small to be considered. This indicated a low probability of inbreeding within the population. And this also showed that the conservation and breeding of Dezhou donkeys in recent years have achieved excellent results. The investigation of genetic diversity in three Dezhou donkey original breeding farms can provide reference data for the selection and breeding of excellent breeds of Dezhou donkey.

## Introduction

**Figure 1:**
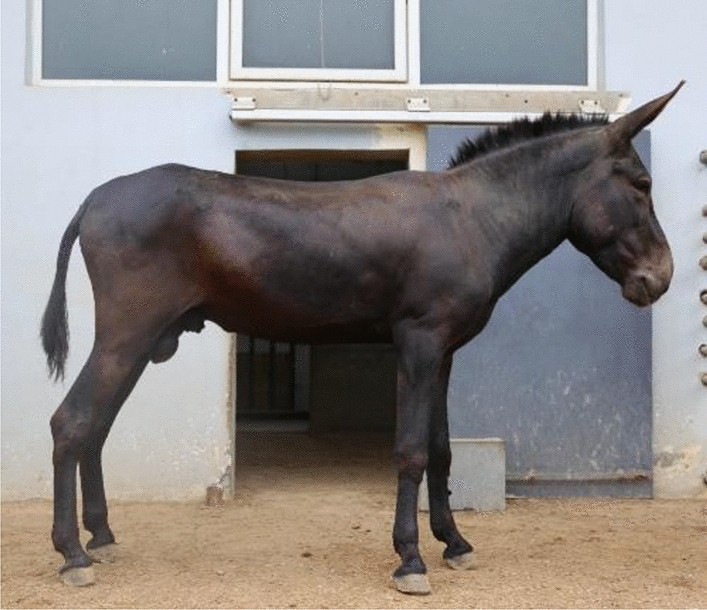
Dezhou donkey.

Dezhou donkey (Fig. 1) is one of the five best donkey breeds in China, with a distinctive feature of tall size, fast growth rate, good production characteristics and stable genetic performance^1,2^. In 2011, Dezhou donkeys were included in the national livestock and poultry genetic resources protection list. Dezhou donkey is an excellent local livestock breed in Shandong Province, China. After careful cultivation, it has gradually formed a unique local breed. With the development of agricultural mechanization, donkeys had gradually lost their traditional role as working animals^3^ and become dual-purpose farm animals^1^. Statistics from China’s Statistical Yearbook showed that the number of donkeys dropped from 9.444 million in 1994 to 2.324 million in 2020. Dezhou donkey breed was facing the problems of rapid population decline and germplasm quality degradation. Therefore, it is urgent to preserve the genetic diversity and variability of Dezhou donkey.

Livestock breeding industry is the foundation of animal husbandry development and the important embodiment of the core competitiveness. Donkeys produce an average of 30 to 90 mL of semen per ejaculation^4^, and according to Shandong Province local standard ‘Technical Specification for Dezhou Donkey Frozen Semen Producing’, a tube of frozen semen for donkeys is 0.5 mL. Therefore, with the application of artificial insemination and other breeding techniques in donkey farms, this resulted in the slaughter of most of the male donkeys at the original breeding farms. Simultaneously, the purchased semen by the breeding farm may not be purebred Dezhou donkeys, which can easily result in crossbreeding with other breeds. This has led to the decline of Dezhou donkey germplasm resources. Therefore, it is very important to investigate the genetic diversity in the Dezhou donkey original breeding farm. At present, the genetic diversity among different Dezhou donkey populations has not been analyzed and reported, this still needs further study.

Microsatellite DNA is widely distributed in chromosomes, which has the advantages of high polymorphism, conservatism, and easy detection^5^. Microsatellite markers have been widely applied to analyze genetic diversity and variability of livestock. In the study of equine species, Zeng et al.^6^ used microsatellite markers to analyze the genetic diversity and structure of Guanzhong horses. Sun et al.^7^ assessed the genetic diversity of donkeys and horses using microsatellite markers. Kefena et al.^8^, Zhang et al.^9^, and Aranguren-méndez et al.^10^ have analyzed the genetic diversity and variability of different donkey breeds. However, the analysis of genetic diversity among different Dezhou donkey populations by microsatellite markers has not yet been done and reported.

In this study, the polymorphism of eight microsatellite loci in Dezhou donkey was investigated by capillary electrophoresis sequencing, and genetic diversity as well as genetic distance in three Dezhou donkey populations were analyzed. This provides the basis for the conservation of biodiversity and genetic resources of the Dezhou donkey.

## Materials and methods

### Moral statement

The experimental animals and methods used in this study were approved by the Animal Policy and Welfare Committee of Liaocheng University (no. LC2019-1). The care and use of laboratory animals were in full compliance with local animal welfare laws, guidelines and policies.

### Sample collection

**Figure 2:**
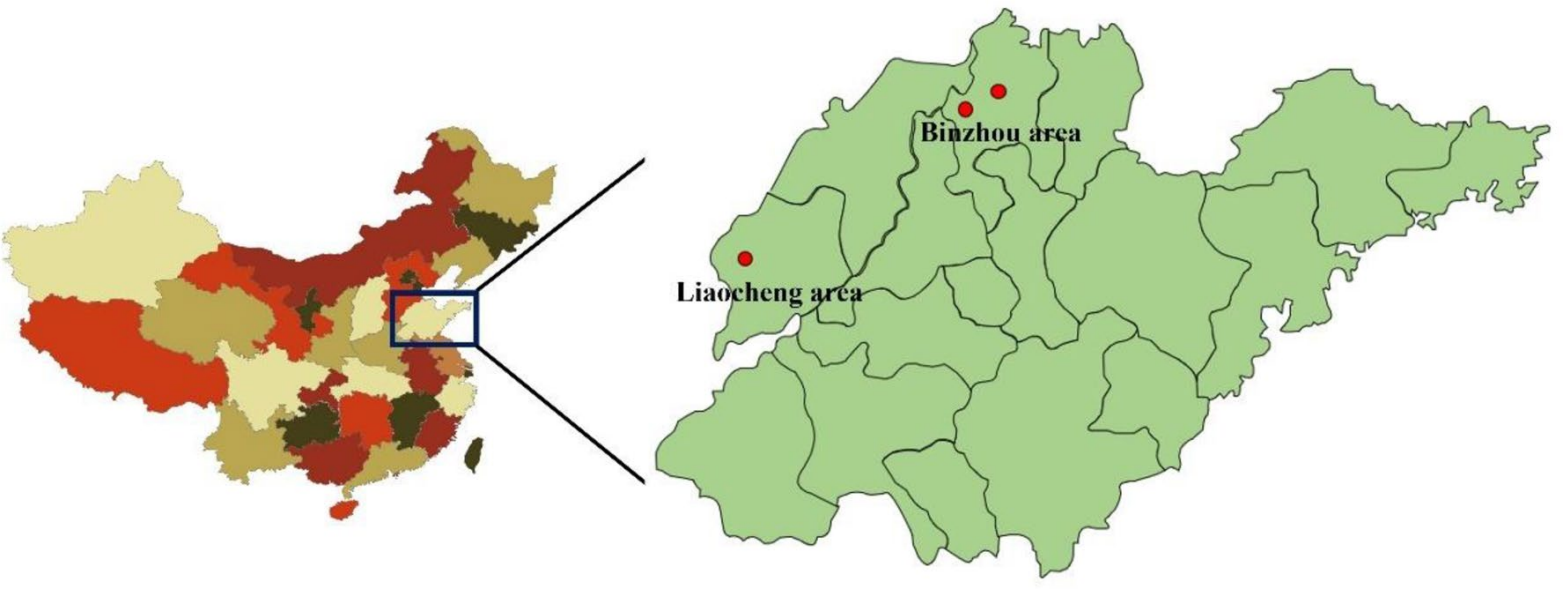
Maps of the Dezhou donkey populations showing the sample locations in the Liaocheng and Binzhou areas in Shandong, China.

Samples were collected from three Dezhou donkey original breeding farms. One of the three breeding farms is located in Liaocheng City, Shandong Province, and two are located in Binzhou City, Shandong Province. A total of 272 individuals were randomly collected from three breeding farms, including 67 individuals in Liaocheng (pop1), 103 individuals in Binzhou 1 (pop2), and 102 individuals in Binzhou 2 (pop3), which represent the major genetic resources of Dezhou donkey. The farm location is shown in Fig. 2. Blood samples were collected from the jugular vein using an EDTA blood collection tube and stored immediately in a – 20 °C refrigerator until use^11^.

### DNA extraction, PCR amplification and Microsatellite genotyping

DNA was extracted from blood using the M5 FlexGen Blood DNA kit (TIANGEN, Beijing, China). The DNA purity (OD260/OD280) was measured by spectrophotometer (B500, Metash, Shanghai, China), and the quantity of genomic DNA extracted was measured using 1% agarose gel^12^.

According to the results of previous studies^13^, eight microsatellite loci were analyzed. The primer sequences are shown in Table S1. The total volume of PCR reaction was 25 μL, including 12.5 µL PCR Mix (Mei5bio, Beijing, China), 8.5 µL ddH_2_O, 1 µL forward primer (Sangon Biotech, Shanghai, China), 1 µL reverse primer (Sangon Biotech, Shanghai, China) and 2 µL genomic DNA^11^. PCR reaction procedure: 35 cycles of predenaturation at 94 °C for 2 min, denaturation at 94 °C for 30 s, annealing for 50 s, and extension at 72 °C for 30 s, it was extended at 72 °C for 40 min and stored at 4 °C^14^.

PCR products were detected on 1% agarose gels, and those with specificity were separated by capillary electrophoresis using an ABI 3730xlDNAAnalyzer (Applied Biosystems, Foster City, CA, USA)^12^, the reagents were as follows: Applied Biosystems POP-7 Polymer and Hi-Di Formamide, running parameters: Run Voltage: 15,000, Injection Voltage: 1600, Injection Duration: 15, Temperature: 63, Laser Power: 12. Fluorescently labeled fragments were detected and sized using GeneMarker version 2.6.4 (Applied Biosystems, USA).

### Population analyses

The Hardy–Weinberg equilibrium (HWE), Observed Heterozygosity (*Ho*), Expected Heterozygosity (*He*), No. Effective Alleles (*Ne*), No. Alleles (*Na*), number of private alleles (NPA) and principal coordinates analysis (PCoA) were calculated using GenAlEx version 6.51^15^. Polymorphic information content (*PIC*), the number of alleles (K), and the number of individuals at the locus (N) were calculated using Cervus version 3.0.7^16^. Percentage of estimated genetic distance from the Fst Values for Dezhou donkeys from the three populations were calculated using GenAlEx version 6.51^15^. Arlequin version 3.1 software was used for AMOVA analysis^17^. Popgene version 1.32 software was used to generate the phylogenetic tree of three Dezhou donkey populations^12^. Mega version 11.0 software and PowerMarker version 3.25 software were used to generate the phylogenetic tree of 272 Dezhou donkeys^18,19^.

### Institutional review board statement

Experiments and animal care in this study were approved by the Animal Welfare and Ethics Committee of Institute of Animal Sciences, Liaocheng University (no. LC2019-1). This study in accordance with the arrival guidelines.

## Results and discussion

### Genotyping results and polymorphism of microsatellite loci

**Figure 3:**
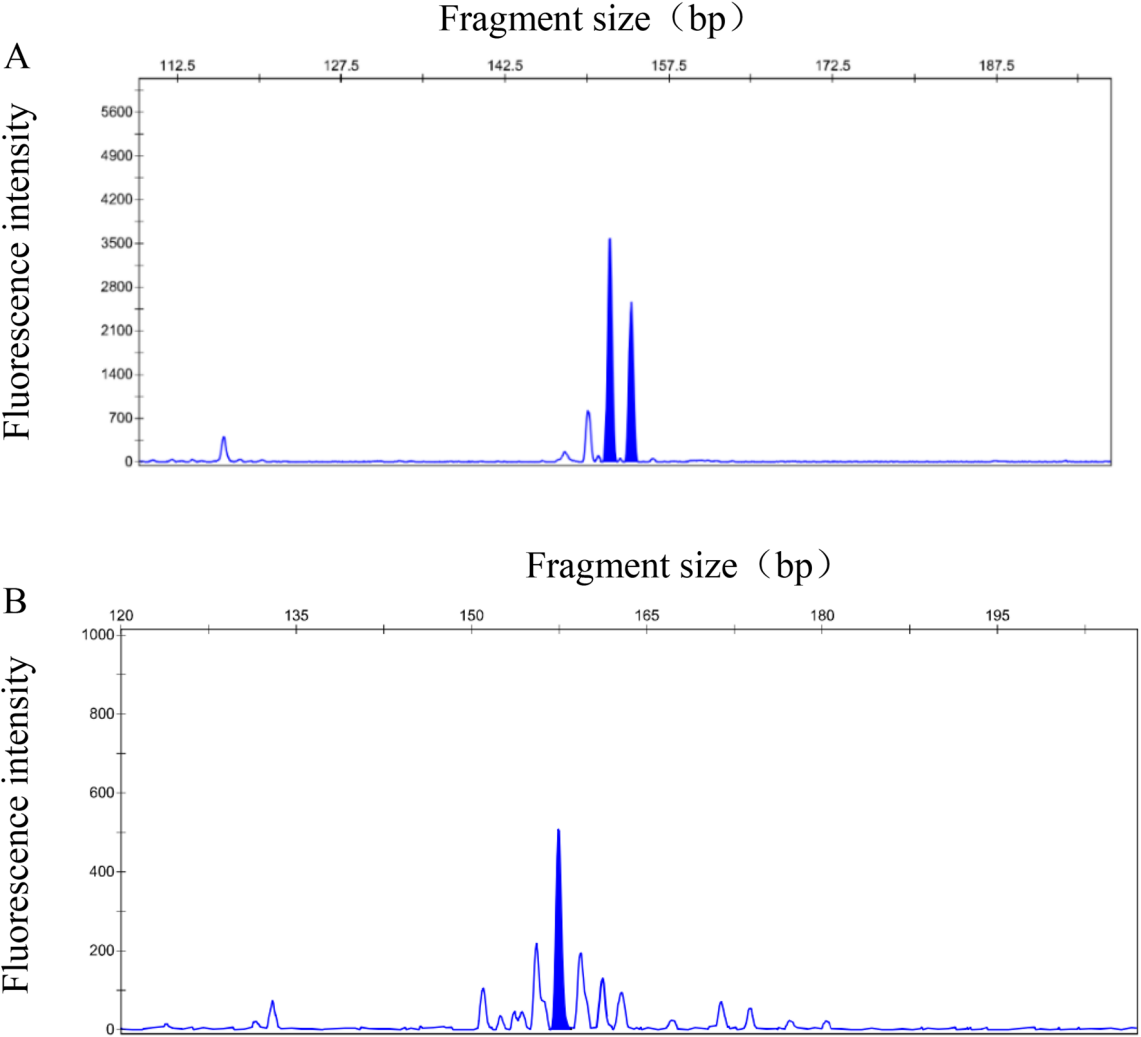
STR detection data reading. (**A**) Shows the genotyped heterozygous peak, and (**B**) shows the genotyped homozygous peak.

Eight microsatellite markers were amplified in three Dezhou donkey populations. Figure 3A shows the genotyped heterozygous peak, and Fig. 3B shows the genotyped homozygous peak. Table 1 shows a summary of the genotyping results. The HMS2 locus had the highest number of alleles, while the HMS6 locus had the lowest number of alleles in pop 1 and pop 3. The TKY343 locus had the highest number of alleles, while the TKY337 locus had the lowest number of alleles in pop 2. The number of alleles at each of the eight loci in pop 2 and pop 3 was higher than that in pop 1, which may be due to the fact that pop 1 had fewer individuals than pop 2 and pop 3. Shang et al.^20^ constructed a set of multiplex PCR systems for horses, which can amplify multiple microsatellite marker loci by a single PCR reaction, with the advantage of saving time and effort, but the study has not been reported by relevant studies on donkeys, and the method needs our further study on donkeys.

**Table1:** Summary of genotyping results.

Pop	Loci	TKY337	HMS7	TKY343	HMS2	HMS6	AHT4	ASB23	HMS3
1	k	5	7	5	12	3	7	6	9
	N	66	64	62	67	67	66	66	67
	PIC	0.479	0.570	0.532	0.832	0.351	0.695	0.763	0.600
	HWE	***	***	ns	ns	***	**	ns	ns
	NPA	0	1	1	2	0	0	6	3
2	k	6	11	18	13	9	10	10	8
	N	103	103	100	103	103	103	103	103
	PIC	0.556	0.620	0.803	0.762	0.600	0.748	0.720	0.627
	HWE	ns	***	***	***	***	ns	***	ns
	NPA	0	2	8	0	4	0	2	0
3	k	8	8	10	12	6	9	12	9
	N	99	102	102	102	102	102	99	102
	PIC	0.617	0.422	0.726	0.705	0.568	0.734	0.782	0558
	HWE	**	***	***	***	***	**	***	*
	NPA	2	0	0	0	1	0	4	0

K: the number of alleles, N: the number of individuals at the locus. *PIC*: polymorphic information content, *PIC* < 0.25, low polymorphism; 0.25 < *PIC* < 0.5, intermediate polymorphism; *PIC* > 0.5, high polymorphism. HWE: Hardy–Weinberg Equilibrium, ns = not significant, *P < 0.05, **P < 0.01, ***P < 0.001. NPA: number of private alleles.

*PIC* is an indicator of gene abundance and its level reflects the diversity of the genetic base of a breed, the *PIC* reflects the genetic variation of microsatellite loci^21^. All eight loci in population 2 were highly polymorphic, all loci except TKY337 and HMS6 were highly polymorphic in pop 1, and all loci except HMS7 were highly polymorphic in pop 3 (*PIC* > 0.5), indicating that they were all highly polymorphic. In this study, abundant polymorphism has been observed for all three populations. Zhang et al.^9^ found that the mean *PIC* for the seven polymorphic loci of the Dezhou donkey population was 0.4561, in the present study the *PIC* results for all three of our populations were higher than that result, this may be due to the large sample size. Consistent with the results of Kim et al.^7^, AHT4, ASB23, HMS2 and HMS3 were highly polymorphic in the donkey population.

Except for HMS7 and HMS6, the remaining loci showed different levels of Hardy–Weinberg equilibrium in the three populations. Four loci (TKY343, HMS2, ASB23, and HMS3) in population 1 were not in Hardy–Weinberg equilibrium, which may be due to the smaller sample size of pop 1 compared with pop 2 and pop 3. All eight loci in pop 3 were in Hardy–Weinberg equilibrium. Pop 2 had the highest number of private alleles, and the TKY343 locus had eight private allele numbers (Table S2). Six private alleles had frequencies greater than 5% (ASB23, 159 bp with a frequency of 28.8%; ASB23, 161 bp with a frequency of 14.4%; ASB23, 165 bp with a frequency of 17.4%; ASB23, 167 bp with a frequency of 25.0%; ASB23, 169 bp with a frequency of 6.8%; HMS3, 153 bp with a frequency of 8.2%). The private alleles with genotype frequencies > 5% were all in pop 1, which may be due to the relatively distant geographical location of pop 1 from population 2 and pop 3. Aranguren-Méndez et al.^10^. found that only one private allele (HTG6) had a frequency greater than 5% in Encartaciones breed. This may be due to the large number of populations selected for this study and the relatively long geographical distance of the populations.

### Genetic diversity of three populations of Dezhou donkey

Genetic variability parameters were calculated for the three populations (Table 2 and Table S3). The mean *Na* for three Dezhou donkey populations was 8.875, ranging from 6.750 (pop1) to 10.625 (pop 2). The *Ne* was the highest in the pop 2 (3.787) and lowest in the pop 1(3.366), with a mean of 3.527. The *Ho* and *He* of pop 2 were the highest, which were 0.646 and 0.717, respectively. The *Ho* and *He* of pop 1 were the lowest, which were 0.498 and 0.646, respectively. Interestingly, these findings were also found in other studies, for example, Aranguren-Méndez et al.^10^ reported an average *He* in 13 microsatellite markers ranging between 0.637 and 0.684 for five endangered Spanish donkey breeds, which is basically consistent with our results. Bordonaro et al.^22^ found that the *He* of the endangered Pantesco and two other Sicilian donkey breeds was lower than that of our study population, ranging from 0.471 for Pantesco to 0.589 for Grigio.

**Table 2:** Genetic variability parameters.

Pop	Sample size	Locality	Na	Ne	Ho	He
Pop1	67	Chiping City, Shandong Province	6.750	3.366	0.498	0.646
Pop2	103	Binzhou City, Shandong Province	10.625	3.787	0.646	0.717
Pop3	102	Binzhou City, Shandong Province	9.250	3.427	0.585	0.678

*Na* No.Alleles, *Ne* No.Effective Alleles, *Ho* observed heterozygosity, *He* expected heterozygosity.

### Genetic distance and relationship among three Dezhou donkey populations

**Figure 4:**
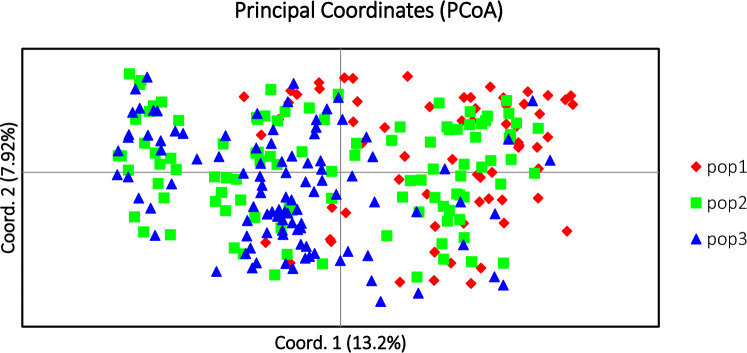
Principal coordinate analysis (PCoA) for three Dezhou donkey populations of China. Coordinates 1 and 2 represent the first two principal coordinates.

The PCoA method was performed to investigate possible genetic relationships among Dezhou donkey populations (Fig. 4). The PCoA analysis demonstrates that samples from the three conservation farms are mixed together. And the PCoA results showed that coordinate 1 (13.2%) mostly separated pop 2 from pop 1. This indicates that pop 1 and pop 2 are relatively genetic distant. However, coordinate 2 (7.92%) did not split the three Dezhou donkey populations into any group. Similarly, Kefena et al.^8^ explored the population structure of native Ethiopian donkeys (Equus asinus) with equine using the PCoA method, coordinate 1 (24.19%) mainly distinguished Abyssinian, Afar and Haraji donkeys from Ogaden, Omo and Sinnar donkeys, coordinate 2 (19.29%) did not separate Ethiopian donkeys into any group, this is consistent with our results.

**Figure 5:**
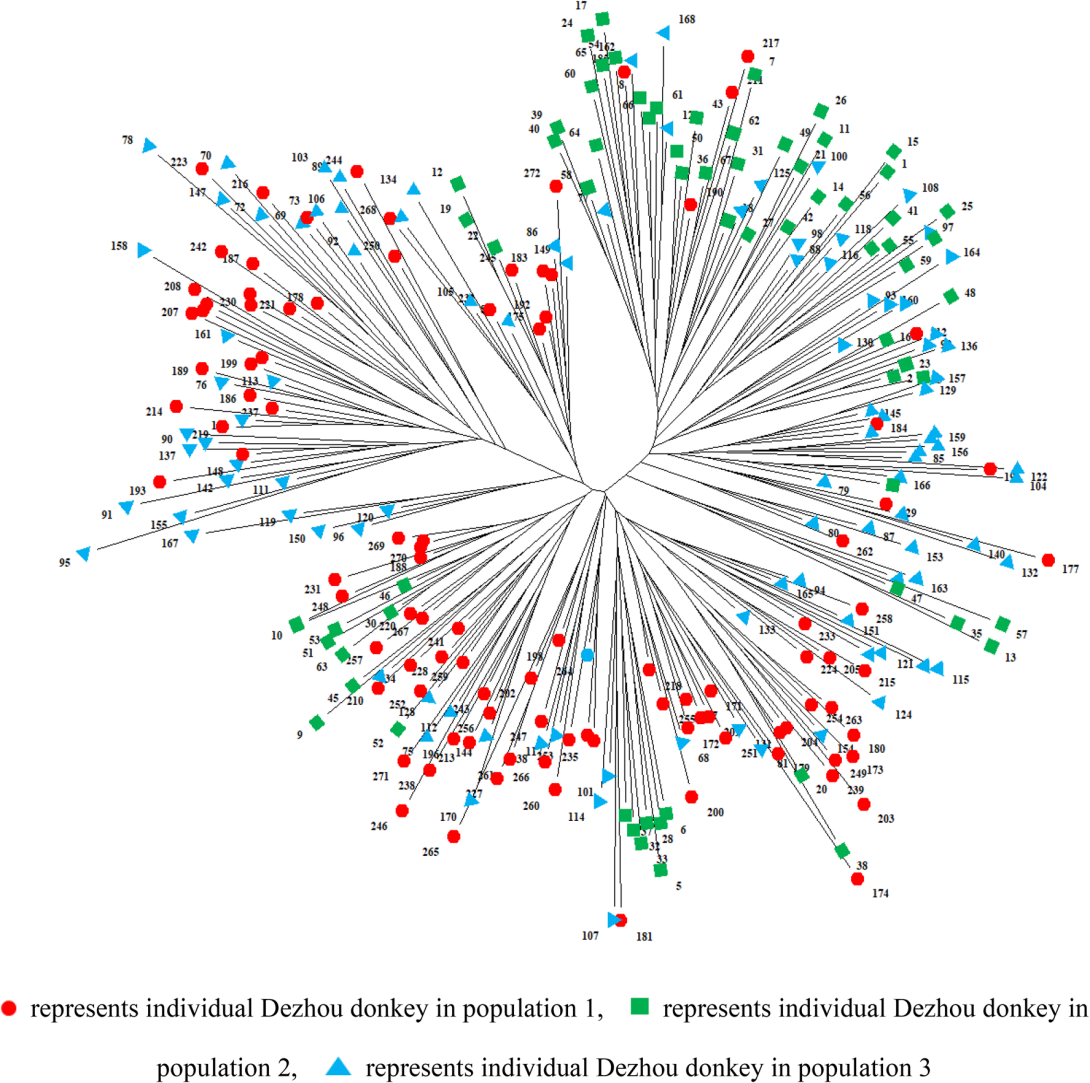
The phylogenetic tree of 272 Dezhou donkeys. The number for each branch is corresponds to each individual of Dezhou donkey.

**Figure 6:**
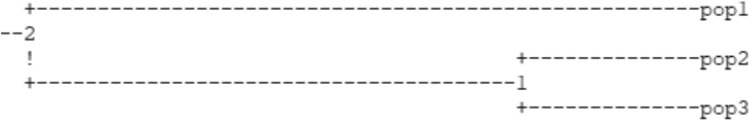
The phylogenetic tree of three Dezhou donkey populations. The number for each branch is corresponds to each population of Dezhou donkey.

Figure 5 shows a phylogenetic tree of 272 Dezhou donkeys from 3 populations that was constructed by using the NJ method. The phylogenetic tree results clustered that the 272 donkeys were divided into 6 groups. Consistent with the PCoA results, pop 2 and pop 3 were closely related. One branch of the phylogenetic tree has only five Dezhou donkeys from pop 3, suggesting that the donkeys may be descended from the same breeding male. Two Dezhou donkey populations, pop 2 and pop 3, clustered together apart from pop 1 (Fig. 6). This may be due to the geographic proximity of pop 2 and pop 3 and the relatively distant geographic location of pop 1 from pop 2 and pop 3. There are only pop 2 and pop 3 on one of the branches of the phylogenetic tree, which indicates that there is gene exchange between pop 2 and pop 3. In the same way, Kim et al.^7^ found that thoroughbreds overlap with Jeju Halla horses in the phylogenetic tree.

The genetic distances according to Nei (1972) are summarized in Table 3. In total, the three Dezhou donkey populations showed high genetic similarity (genetic distances between 0.053 and 0.229). The genetic distance between pop 3 and pop 1 and the genetic distance between pop 2 and pop 1 were higher than those between pop 1 and pop 2. This is consistent with the phylogenetic tree results. Wang et al.^10^ found that the genetic distance of the Spanish donkey breeds ranged from 0.057–0.093, this is lower than our results.

**Table 3:** Pairwise population matrix of Nei genetic distance.

Population	Pop1	Pop2	Pop3
Pop1	0.000		
Pop2	0.167	0.000	
Pop3	0.229	0.053	0.000

The AMOVA analysis is a satisfactory grouping criterion for evaluating the variation within and among populations^23^. AMOVA analysis showed that 0.14382 of the genetic variation occurred among populations and 2.70564 of the genetic variation occurred within the population, indicating that the genetic variation was mainly concentrated within the population and the genetic differentiation among the populations was low (Table 4). The Fst values of pop 1 and pop 2, pop 1 and pop 3, pop 2 and pop 3 are all in 0–0.05 (Table 5). When the Fst value is 0–0.05, it indicates that the genetic differentiation among populations is very small and cannot be considered. Genetic structure coefficients, as described by F-statistics, are presented in Table 6. Three loci (HMS7, ASB23 and TKY343) had Fst values in the range of 0.05–0.15, which indicated a moderate degree of genetic differentiation between populations. Although the Fst values of three loci (HMS7, ASB23 and TKY343) ranged from 0.05 to 0.15, the Fst values of pop 1 and pop 2, pop 1 and pop 3, and pop 2 and pop 3 all ranged from 0 to 0.05. The Fis value of the HMS3 locus was negative (− 0.0006), indicating a low level of inbreeding. This indicates a low probability of inbreeding within the population, and breeders still need to take appropriate measures to protect the Dezhou donkey germplasm. This also shows that the conservation and breeding of Dezhou donkeys in recent years have achieved excellent results. The conservation farms are managed correctly, and breeders still need to take appropriate measures to protect the Dezhou donkey germplasm.

**Table 4:** Analysis of molecular variance for three genetic groups of Dezhou donkey based on allele frequencies from 8 microsatellite markers.

Source of variation	d.f	Sum of squares	Variance component	Percentage of variation
Among populations	2	56.680	0.14382	5.05
Within populations	541	1463.752	2.70564	94.95
Total	543	1520.432	2.84946	

*d.f.* degrees of freedom.

**Table 5:** Percentage of estimated genetic distance from the F_ST_ Values for Dezhou donkeys from the three populations.

Population	Pop1	Pop2	Pop3
Pop1	0.000		
Pop2	0.033	0.000	
Pop3	0.049	0.013	0.000

**Table 6:** F-Statistics over All Pops for each Locus.

Pop	Locus	Fis	Fit	Fst
All pops	TKY337	0.293	0.308	0.021
	AHT4	0.086	0.098	0.013
	HMS6	0.293	0.312	0.027
	HMS2	0.030	0.042	0.013
	HMS7	0.589	0.632	0.105
	ASB23	0.018	0.129	0.113
	HMS3	− 0.006	0.006	0.013
	TKY343	0.071	0.092	0.023
	Mean ± SE	0.172 ± 0.073	0.203 ± 0.073	0.041 ± 0.015

*Fis* deviation from Hardy-Weinberg proportions(within-population inbreeding coefficient); *Fit* inbreeding coefficient of an individual relative into the whole population; *Fst* coefficient of differentiation, fixation index.

## Conclusions

In conclusion, we evaluated genetic diversity in three Dezhou donkey breeding farms using microsatellite markers. The results showed that abundant polymorphism and low inbreeding levels were observed in all three populations, this provides a direction for the conservation of genetic diversity of Dezhou donkeys. However, this study was limited by the low sample size, and further investigation is needed to expand the sample size in the future.

## Supplementary Information

Supplementary Tables.

## Data Availability

The data presented in this study are available on request from the corresponding author.
